# Exploring BMI and DMFT Indexes of Male Islamic Boarding School Students in Indonesia: A Cross-Sectional Study

**DOI:** 10.34172/joddd.44130

**Published:** 2026-03-30

**Authors:** Laifa Annisa Hendarmin, Muhammad Faiza Ma'ruf, Risahmawati Risahmawati, Witri Ardini, Sarah At Tauhidah

**Affiliations:** ^1^Department of Medical Biology, Faculty of Medicine, Universitas Islam Negeri Syarif Hidayatullah Jakarta, Banten, Indonesia; ^2^Faculty of Medicine, Universitas Islam Negeri Syarif Hidayatullah Jakarta, Banten, Indonesia; ^3^Department of Community Medicine, Faculty of Medicine, Universitas Islam Negeri Syarif Hidayatullah Jakarta, Banten, Indonesia; ^4^Department of Clinical Nutrition, Faculty of Medicine, Universitas Islam Negeri Syarif Hidayatullah Jakarta, Banten, Indonesia

**Keywords:** Adolescent, Body mass index, DMFT index, Dental caries, Islamic boarding school, Students

## Abstract

**Introduction:**

Adolescent health reflects both nutritional and oral conditions, which share dietary and behavioral risk factors. In Muslim societies, Islamic boarding schools (*pesantren*) shape adolescent lifestyles through communal meals, structured routines, and religious values of cleanliness. Their students, called *santri*, represent a unique adolescent population. This study examined the relationship between the body mass index (BMI) and dental caries experience, measured by the decayed, missing, and filled teeth (DMFT) index, among male santri in Indonesia.

**Methods:**

A cross-sectional study was conducted in 2024 among 85 male santri at an Islamic boarding school in Bogor, Indonesia. BMI was classified according to CDC-2000 growth charts. Dental caries experience was assessed using the WHO DMFT index. Descriptive statistics summarized BMI and DMFT distributions. Associations were evaluated using the chi-squared or Fisher’s exact test.

**Results:**

Most participants had normal BMI (65.9%), while 18.8% were overweight, and 10.6% were obese. Regarding oral health, 55.3% had very low DMFT scores, and 10.6% had high or very high caries experience. No significant association was observed between BMI and DMFT categories (*P*=0.276).

**Conclusion:**

Although no significant association was identified, the coexistence of obesity and high caries in a subset of santri indicates a dual public health concern. These findings warrant further investigation and highlight the need for preventive strategies addressing both nutrition and oral health in Islamic boarding school settings.

## Introduction

 Oral health is an integral component of general health and well-being. The World Health Organization (WHO) Global Oral Health Status Report (2022) highlighted that oral diseases, particularly dental caries, remain a major public health challenge worldwide, affecting more than 2 billion people, over one third of the global population.^1^ In Indonesia, the 2018 Basic Health Research Survey (Riskesdas) reported that 88.8% of the population had experienced dental caries, reflecting a very high national burden.^2^ Caries prevalence increases gradually from adolescence into adulthood, making early prevention a critical public health priority.^3^

 The DMFT index is a standardized measure widely used in epidemiological studies to assess cumulative caries experience.^4^ Numerous studies have demonstrated that knowledge, behavior, and attitudes toward oral health influence DMFT outcomes.^5-7^ For example, Indonesian studies among university students and schoolchildren reported significant knowledge‒DMFT associations,^7,8^ and findings from Saudi Arabia further highlight how population-level factors relate to caries patterns.^9^

 At the same time, nutritional status, often assessed using the body mass index (BMI), has been increasingly recognized as an important determinant of oral health. Both obesity and undernutrition share common dietary risk factors with dental caries, particularly excessive sugar consumption and poor diet quality.^10^ However, the relationship between BMI and caries remains inconsistent across studies and may be influenced by contextual and cultural factors, including hygiene practices, dietary environment, and access to care.^11^

 In Muslim societies, Islamic boarding schools (*pesantren*) provide a unique living and learning environment where adolescents (*santri*) spend extended periods in communal settings. Their daily lives are shaped by structured routines, shared diets, and religious teachings emphasizing cleanliness (*ṭ ahārah*). These distinctive conditions may influence both nutritional status and oral health; yet, little is known about the interplay between BMI and dental caries in this population.

 This study aimed to examine the relationship between BMI and DMFT index among male adolescents in an Islamic boarding school in Indonesia. By focusing on this under-researched population, the study provides insights into the dual public health challenges of nutrition and oral health in culturally specific educational settings.

## Methods

###  Study Design and Participants

 This cross-sectional study was conducted in 2024 among male students in an Islamic boarding school in Bogor, Indonesia. From an initial pool of 100 students, 85 met the inclusion criteria (adolescents aged 14‒17 years, free from systemic diseases affecting growth or oral health, and provided consent to participate). Exclusion criteria included incomplete data, systemic illness, or refusal to participate. A consecutive sampling approach was used. Only male students were included because male and female students in Islamic boarding schools live separately, with distinct environments and routines that could influence health behaviors.

###  Anthropometric Measurement

 Body weight and height were measured using calibrated instruments. BMI was calculated as weight (kg) divided by height squared (m^2^) and categorized according to the CDC-2000 growth charts into underweight ( < 5th percentile), normal (5th‒85th percentile), overweight (85th‒95th percentile), and obese ( ≥ 95th percentile).^12^

###  Dental Examination

 Oral examinations were performed by a trained dentist using disposable instruments under adequate light. Caries experience was assessed according to WHO criteria for the DMFT index.^4^ DMFT scores were further categorized into very low (0.0‒1.1), low (1.2‒2.6), moderate (2.7‒4.4), high (4.5‒6.5), and very high ( ≥ 6.6), based on WHO classification.^4^

###  Statistical Analysis

 Data were analyzed using SPSS 24.0 (IBM Corp., Armonk, NY, USA). Descriptive statistics were presented as frequencies and percentages. Associations between BMI and DMFT categories were tested using the chi-squared/Fisher’s exact test, as appropriate. Statistical significance was set at *P* < 0.05.^13^

## Results

###  Participant Characteristics

 Eighty-five male students participated in the study (Table 1). The largest age group was 16 years (42.4%), followed by 15 years (35.3%), 17 years (20.0%), and 14 years (2.4%). Based on BMI, 65.9% were categorized as normal, 18.8% as overweight, 10.6% as obese, and 4.7% as underweight.

**Table 1:** Demographic characteristics of participants (n = 85)

**Variable**	**n**	**%**
Age (years)		
14	2	2.4
15	30	35.3
16	36	42.4
17	17	20.0
BMI category		
Underweight ( < 5th percentile)	4	4.7
Normal (5th–85th percentile)	56	65.9
Overweight (85th–95th percentile)	16	18.8
Obese ( ≥ 95th percentile)	9	10.6

BMI categorized according to CDC-2000 growth charts.

###  Dental Caries Distribution

 The majority of students (55.3%) had very low DMFT scores, followed by 15.3% with low, 15.3% with moderate, 7.1% with high, and 7.1% with very high DMFT scores (Table 2).

**Table 2:** Distribution of DMFT scores among participants (n = 85)

**DMFT Category**	**n**	**%**
Very low (0.0–1.1)	47	55.3
Low (1.2–2.6)	13	15.3
Moderate (2.7–4.4)	13	15.3
High (4.5–6.5)	6	7.1
Very high ( ≥ 6.6)	6	7.1

DMFT categories based on WHO classification

###  Association between BMI and DMFT

**Figure 1:**
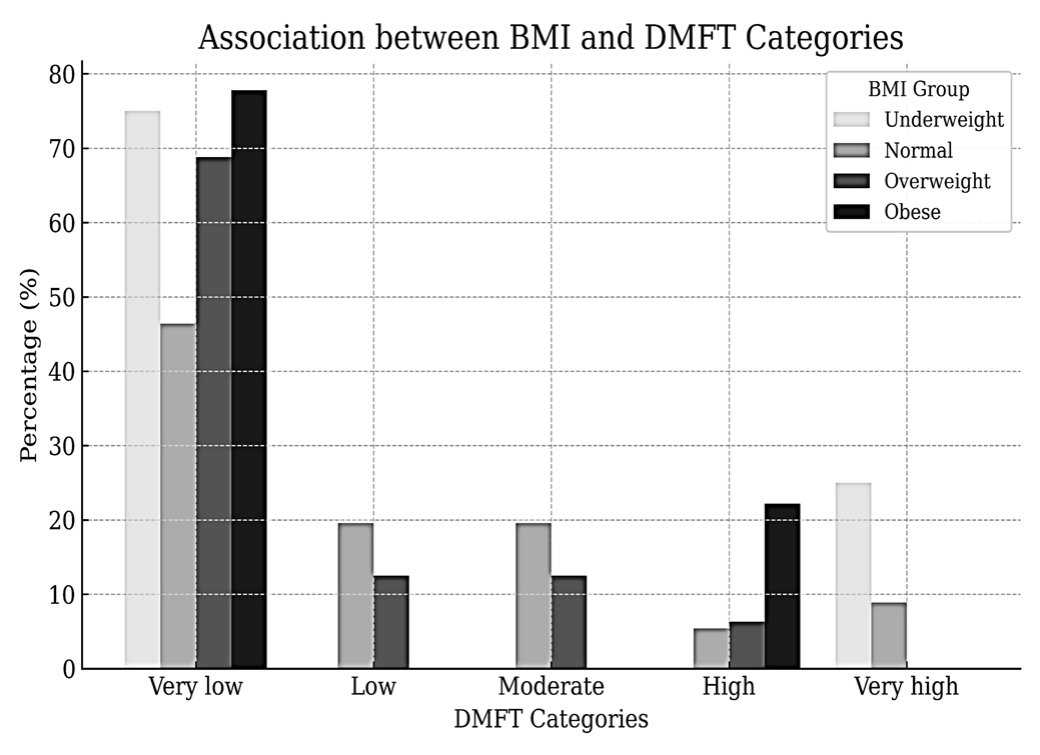
Association between BMI and DMFT categories among adolescent male students at Islamic Boarding School (n = 85). Percentages within each BMI group are shown for five DMFT categories (very low, low, moderate, high, very high). No significant association was observed. (chi-squared test, *P* = 0.276).

 Cross-tabulation showed that overweight and obese students tended to cluster in the very low and high DMFT categories; however, the association between BMI and DMFT was not statistically significant (*P* = 0.276) (Table 3, Figure 1).

**Table 3:** Association between BMI and DMFT categories (n = 85)

**BMI group**	**Very low n (%)**	**Low n (%)**	**Moderate n (%)**	**High n (%)**	**Very high n (%)**	***P*** value*
Underweight	3 (75.0)	0 (0)	0 (0)	0 (0)	1 (25.0)	0.276
Normal	26 (46.4)	11 (19.6)	11 (19.6)	3 (5.4)	5 (8.9)	
Overweight	11 (68.8)	2 (12.5)	2 (12.5)	1 (6.3)	0 (0)	
Obese	7 (77.8)	0 (0)	0 (0)	2 (22.2)	0 (0)	

Chi-squared/Fisher’s Exact test.

## Discussion

 This study examined the relationship between BMI and dental caries experience (DMFT index) among male students in an Islamic boarding school in Indonesia. The majority of participants had a normal BMI, while approximately one-third were overweight or obese. More than half showed very low DMFT scores, although 10.6% presented with high or very high caries experience. There was no significant association between BMI and DMFT.

 The absence of a significant relationship is consistent with findings from several previous studies. A systematic review by Paisi et al.^14^ reported that among higher-quality studies, most found no association between BMI and dental caries, although some observed positive correlations in older children. Likewise, a recent adolescent pilot study found that obesity defined by BMI was not associated with caries activity.^15^ Similarly, research among adolescents by Lopez del Valle et al.^16^ reported no association between BMI and caries.By contrast, population-based data from Ecuador suggested a more complex, non-linear BMI‒caries pattern without an overall significant association; potential behavioral and environmental influences warrant further studies.^17^

 Other investigations further emphasize inconsistency. A meta-analysis by Hayden et al.^11^ highlighted significant heterogeneity across populations.Regional data also illustrate variability: research in Saudi Arabia showed differences in caries burden related to environmental context such as water fluoride levels,^18^ while school-based studies in other populations have reported positive BMI‒caries correlations.^19,20^ In addition, lifestyle and dietary risk exposures remain important correlates of caries: an analysis in Malaysian preschoolers showed associations with nutritional status, sugar intake, and second-hand smoke exposure.^21^

 The relatively uniform environment of Islamic boarding schools may help explain the absence of a significant association in this study. Students typically share diets, routines, and hygiene practices, which could reduce variability in both BMI and DMFT outcomes. Nevertheless, the coexistence of obesity and high caries in a subset of students highlights the dual burden of malnutrition and oral disease in Indonesia.^2^ Poor dietary habits, particularly excessive sugar intake, contribute to both conditions and reinforce the need for integrated prevention.^3,21^

 Gender-specific factors are also relevant. This study included only male students, reflecting the gender-segregated structure of many Islamic boarding schools. Female students often demonstrate more favorable oral hygiene behaviors, which may result in different caries outcomes.^22,23^ Focusing on a male-only sample reduced potential confounding due to gender-related behavioral differences, but future research should include female cohorts to provide a more comprehensive understanding.

 Overall, these findings emphasize that the relationship between BMI and caries cannot be explained by nutritional status alone. Broader determinants including oral hygiene practices, dietary behaviors, and socio-environmental influences likely play an important role. Boarding schools, with their communal living and strong cultural values, present an opportunity for integrated interventions that combine nutrition education with oral health promotion, aligned with religious teachings on cleanliness and moderation.

## Limitations

 This study has several limitations. It was conducted in a single institution with a relatively small and homogenous sample, which may limit generalizability. The cross-sectional design precludes causal inference. Important confounders such as diet, oral hygiene practices, and socioeconomic background were not assessed. Furthermore, the DMFT index, while widely used in epidemiological surveys, does not distinguish between active and inactive caries or capture early enamel lesions, which may have led to underestimation of caries activity in the study population.

## Conclusion

 No significant association between BMI and DMFT was observed among male students in an Islamic boarding school in Indonesia. However, the coexistence of obesity and high caries among a subset of students reflects a dual public health concern. These findings highlight the importance of school-based programs that integrate balanced diet education with daily supervised toothbrushing and culturally tailored oral health promotion to improve adolescent well-being in boarding school settings.

## Competing Interests

 The authors declare no conflicts of interest related to this work.

## Data Availability of Statement

 The datasets generated and analyzed during the current study are available from the corresponding author upon reasonable request.

## Ethical Approval

 This study received approval from the Health Research Ethics Committee, Faculty of Medicine, Universitas Islam Negeri Syarif Hidayatullah Jakarta (No. B-040/F12/KEPK/TL.00/11/2024). Written informed consent was obtained from all participants and their guardians.
